# Protective Effect and Mechanism of Bone Morphogenetic Protein-4 on Apoptosis of Human Lens Epithelium Cells under Oxidative Stress

**DOI:** 10.1155/2021/8109134

**Published:** 2021-01-29

**Authors:** Bei Du, Jia-Lin Zheng, Liang-Yu Huang, Hong Zhang, Qiong Wang, Ya-Ru Hong, Xiao-min Zhang, Xiao-Rong Li, Li-Jie Dong

**Affiliations:** ^1^Tianjin Key Laboratory of Retinal Functions and Diseases, Eye Institute and School of Optometry, Tianjin Medical University Eye Hospital, Tianjin 300384, China; ^2^Eye Hospital, Nanjing Medical University, Nanjing, China; ^3^First Teaching Hospital of Tianjin University of Traditional Chinese Medicine, Tianjin, China

## Abstract

Bone morphogenetic proteins (BMPs), a member of the transforming growth factor *β* (TGF-*β*) superfamily, are abundant in human ocular tissues and play an important role in lens development. Targeted deletion of BMP-4 in mice results in failure of lens placode formation. Following lens maturation, the formation of senile cataracts is demonstrably associated with free radical-related oxidative stress. Previous studies reported that BMPs play an antiapoptotic role in cells under oxidative stress, and the BMP-4 signal is important in inflammation regulation and homeostasis. BMP-4 evidently suppressed the apoptosis of human lens epithelial cells (HLECS) under oxidative stress induced by H_2_O_2_. This protective antiapoptotic effect is partly due to a decrease in caspase-3 activity and reactive oxygen species (ROS) level. Furthermore, the expression of activating transcription factor- (ATF-) 6 and Krüppel-like factor- (KLF-) 6 increased under oxidative stress and decreased after BMP-4 treatment.

## 1. Introduction

Cataracts are a widely prevalent eye disease, which are the leading cause of blindness worldwide and involve a complicated pathogenesis. It is generally accepted that the main mechanism of cataract onset is oxidative damage [1, 2], and the molecular mechanism of cataract pathogenesis has long been a research hotspot.

The bone morphogenetic protein (BMP) is a multifunctional growth factor belonging to the transforming growth factor *β* (TGF-*β*) superfamily that has been shown to play important roles in both the development and regeneration of different tissues [3]. Previous research has shown that BMPs and their receptors play an important role in the development of lens during eyeball development [4]. BMPs are highly expressed in mouse embryos, and blocking of BMP signals in the lens ectoderm of cultured mouse embryos prevented lens formation. The BMP inhibitor Noggin, when added to chick lens epithelial cells, results in abnormal development of the lens [5]. BMP-4 is a member of the BMP family, and targeted deletion of BMP-4 in mice impairs lens placode formation [6]. Moreover, the absence of BMP-4 in mice can lead to irregular turbidity or white patches in the vitreous bodies [7]. Previous studies have reported that BMPs play an antiapoptotic role in some cells under oxidative stress, and the BMP-4 signal is important in the regulation of inflammation and homeostasis [8, 9]. However, limited information is available regarding the role and mechanism of BMP-4 in human lens.

## 2. Materials and Methods

### 2.1. Cell Culture

The human lens epithelial cell line HLE-B3 was obtained from laboratories of the Tianjin Medical University Eye Hospital (Tianjin, China) and incubated in Dulbecco's Modified Eagle Medium (DMEM) with 10% fetal bovine serum (FBS; Gibco, USA), 100 U/mL penicillin, and 100 U/mL streptomycin at 37°C in a humidified atmosphere of 5% CO_2_. The cells were cultured on a 96-well plate following the normal procedure and divided into the control group, H_2_O_2_ group, and H_2_O_2_+BMP-4 group.

For H_2_O_2_-induced oxidative stress, the cells were cultured following the routine procedure. The medium of each group was replaced with serum-free medium for 16 h, then cells were exposed to 300 *μ*M H_2_O_2_ and incubated at 37°C for 1.5 h. Then, serum-free medium containing BMP-4 (100 ng/mL) was added to the H_2_O_2_+BMP-4 group. All experiments were performed in triplicate.

### 2.2. Cell Counting Kit-8 (CCK-8)

The cell suspension was inoculated in groups of 200 *μ*L in a 96-well plate with 5 × 10^3^ cells/well; the plate was then placed in a cell incubator with 5% CO_2_ at 37°C. After cell attachment, H_2_O_2_-induced oxidative stress stimulation was performed using the method mentioned above. Then, the medium was replaced with serum-free medium containing varying concentrations of BMP-4, and the cells were incubated for 24 or 48 h.

The culture medium of the corresponding detection well plate was removed and washed with phosphate-buffered saline (PBS; Gibco, USA). Afterwards, a fresh blank of 100 *μ*L DMEM and 10 *μ*L CCK-8 reagent (Dojindo, Kyushu, Japan) was added into each well, then the culture plate was placed in an incubator with 5% CO_2_ at 37°C for 2 h. A microplate reader was used to measure the cell optical density (OD) value at 450 nm.

### 2.3. Flow Cytometric Analysis of Apoptosis

The HLE-B3 cells were prestimulated with 100 ng/mL BMP-4 in serum-free medium for 2 h, then 300 *μ*M/L H_2_O_2_ was added for 12 h. Next, serum-free medium containing 100 ng/mL BMP-4 was added for 24 h. Then, the HLE-B3 cells were collected and washed with PBS and subjected to a PI/Annexin V FITC Apoptosis Detection kit (CoWin Biosciences, Beijing, China): Briefly, each sample was diluted in 100 *μ*L Annexin binding buffer and then was stained with 5 *μ*L Annexin V-fluorescein isothiocyanate and 5 *μ*L propidium iodide (PI) for 15 min at room temperature in the dark. Following incubation, the cells were analyzed with a FACSCalibur flow cytometer (BD Biosciences, San Diego, CA, USA). Flow cytometric analysis was performed in triplicate.

### 2.4. Analysis of Mitochondrial Membrane Depolarization

The change in the mitochondrial membrane potential (*ΔΨ*m) in HLE-B3 cells was monitored using the mitochondrial membrane potential detection kit (JC-1, T4069, Sigma-Aldrich) according to the manufacturer's instructions. Briefly, HLE-B3 cells cultured in a 96-well plate (5 × 10^3^ cells per well) were treated with Tat followed by treatment with 1x JC-1 reagent diluted in serum-free DMEM for 20 min at 37°C in a 5% CO_2_ atmosphere. Thereafter, cells were rinsed once with 1x rinsing buffer provided with the kit. Fluorescence was measured using the FL600 fluorescent plate reader (BioTek Instruments, Winooski, VT, USA) at excitation wavelengths of 485 and 535 nm. All experiments were repeated at least three times.

### 2.5. Detection of ROS

After 100 ng/mL serum-free BMP-4 was added to the cells for 2 h, they were stimulated for 1.5 h with 300 *μ*M/L H_2_O_2_. The cells were then cultured again in serum-free medium containing 100 ng/mL BMP-4 for 2 h. The Image-iT™ LIVE Green Reactive Oxygen Species (ROS) Detection Kit obtained from Invitrogen (Thermo Fisher Scientific) was used to estimate the ROS level in live HLE-B3 cells. Following treatment of cells according to the experimental conditions, cells were incubated with 15 mM dichlorodihydrofluorescein diacetate (DCFH-DA) (Sigma-Aldrich) for 45 min, briefly centrifuged to remove the dye, and resuspended in 4-(2-hydroxyethyl)-1-piperazineethanesulphonic acid (HEPES) buffer (Thermo Fisher Scientific). The change in fluorescence was measured in a spectrofluorometer set at 485 nm excitation and 530 nm emission. Change in fluorescence intensity was represented in arbitrary units.

### 2.6. Caspase-3 Measurement

The cells were routinely treated as mentioned above. The activity of caspase-3 in cells was measured using a caspase-3 activity kit according to the manufacturer's protocol (BioVision Inc., Milpitas, CA, USA). In brief, cytosolic proteins (200 *μ*g in 50 *μ*L) were mixed with the caspase-3-specific substrate Ac-DEVD-pNA (Jiancheng, Nanjing, China) and incubated at 37°C for 4 h. The absorbance was measured at 405 nm with an enzyme marker.

### 2.7. Reverse Transcription-Polymerase Chain Reaction (RT-PCR) Analysis

The mRNA expression of activating transcription factor- (ATF-) 6 and Krüppel-like factor- (KLF-) 6 in HLE-B3 cells was analyzed using an ABI 7500 real-time PCR system (Applied Biosystems, Foster City, CA, USA). The cells were collected and examined by RT-PCR. The sequences of the primers used for the PCR are listed in Table 1.

### 2.8. Statistical Analysis

SPSS 20.0 statistical software (IBM, USA) was used for statistical analysis. The data of each group were normally distributed by the Shapiro-Wilk test and expressed as mean ± standard deviation. One-way ANOVA was used for comparing cell proliferation rates in different groups, and the Tukey test was used for pairwise comparison between groups. Two-factor ANOVA was used for the overall comparison of each cell group at different time points, and the Tukey test was used for intergroup comparison. The significance level was chosen as *p* < 0.05.

## 3. Results

### 3.1. Effects of BMP-4 on Human Lens Epithelium Cells under Oxidative Stress

**Figure 1:**
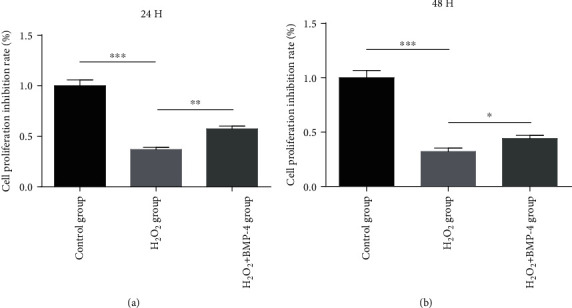
Effects of BMP-4 on human lens epithelium cell under H_2_O_2_-induced oxidative stress. Under oxidative stress, cell proliferation was inhibited significantly, but BMP-4 showed a significant protective effect on cells under oxidative stress. ∗∗ represents *p* < 0.01 vs. H_2_O_2_ group and ∗∗∗ represents *p* < 0.001 vs. H_2_O_2_ group.

Under the oxidative stress of H_2_O_2_ (300 *μ*M/L, 1.5 h), cell proliferation was significantly inhibited compared to that in the control group (*p* < 0.01). Cells were then stimulated with serum-free BMP-4 (100 ng/mL) for 24 and 48 h. The proliferation of BMP-4-treated cells was significantly increased compared to that in the H_2_O_2_ group (*p* < 0.001) (Figure 1).

### 3.2. Changes in HLECSs Assessed by Light Microscopy

The cultured cells were stimulated with H_2_O_2_ for 1.5 h, then 100 ng/mL BMP-4 in serum-free medium was added for 24 h.

**Figure 2:**
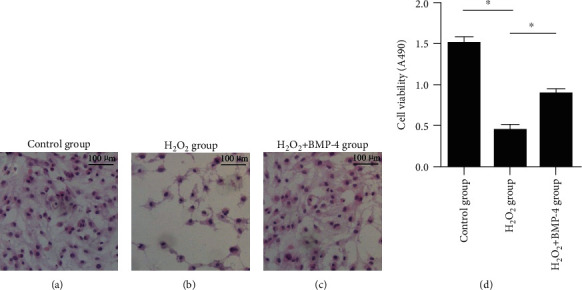
HE staining of cells. Images (a–c) show that the number of HE-labelled cells decreased under oxidative stress by H_2_O_2_ and was markedly increased by the action of BMP-4. (d) Quantification of cell number. Data are expressed as mean ± SEM, ^∗^*p* < 0.05.

Cells in the different groups were stained with haematoxylin-eosin (HE) and observed under light microscopy (Figures 2(a)–2(c)). The micrographs show that normal cells stained by HE were densely packed; however, under oxidative stress by H_2_O_2_, the number of HE-labelled cells decreased. After BMP-4 treatment, the number of cells increased significantly.

**Figure 3:**
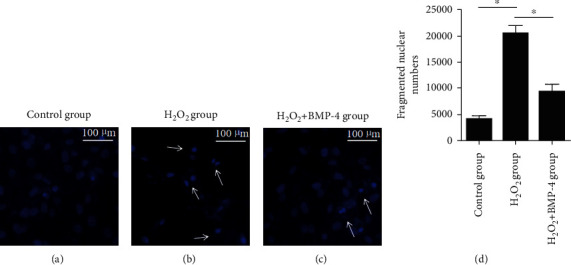
Hoechst staining of cells. Images (a–c) show alterations in the nuclear morphology after H_2_O_2_ and H_2_O_2_+BMP-4 treatment of HLE-B3 cells. Arrows indicate the alterations in nuclear morphology. Normal cell nuclei were stained lightly and uniformly. Under oxidative stress, the nuclei were fragmented and stained with dense hyperchromatism. After BMP-4 treatment, the nucleus fragmentation was obviously improved. (d) Quantification of fragmented nuclear number. Data are expressed as mean ± SEM, ^∗^*p* < 0.05.

Hoechst 33258-2 was used to stain the nuclei of cells (Figures 3(a)–3(c)). The nuclei of normal untreated cells were stained lightly and uniformly by Hoechst 33258-2; however, under oxidative stress by H_2_O_2_, the nuclei were fragmented and appeared loosely packed. After BMP-4 treatment, the nucleus fragmentation was markedly improved.

### 3.3. The Effect of BMP-4 on H_2_O_2_-Induced Apoptosis, ER (Endoplasmic Reticulum) Stress, Increased Caspase-3 Level, and ROS in HLECSs

#### 3.3.1. The Apoptosis of HLECSs under H_2_O_2_ Oxidative Stress

**Figure 4:**
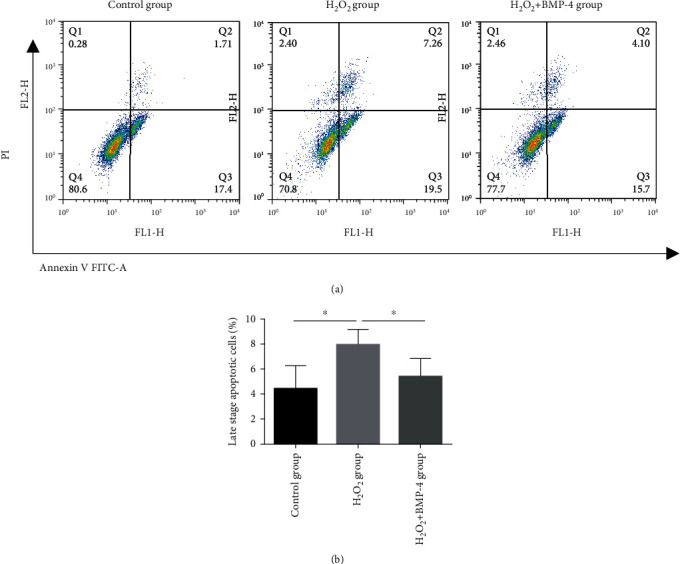
Using flow cytometry analysis to detect the apoptosis of HLECSs: (a) representative images of Annexin V/PI uptake by HLECSs; (b) relative percentage of apoptotic cells was quantified.

After exposure to 100 ng/mL serum-free BMP-4 for 2 h, HLECSs were stimulated for 12 h with 300 *μ*M/L H_2_O_2_ to enter apoptosis and then were cultured again in serum-free medium containing 100 ng/mL BMP-4 for 24 h. The apoptotic cells were detected by flow cytometry. As seen in Figures 4(a) and 4(b), when compared with the control group, apoptosis in the H_2_O_2_ group was significantly increased, whereas the percentage of apoptotic cells in the H_2_O_2_+BMP-4 group was significantly reduced compared with that in the H_2_O_2_ group, which indicated that BMP-4 could inhibit H_2_O_2_-induced apoptosis of HLECSs.

**Figure 5:**
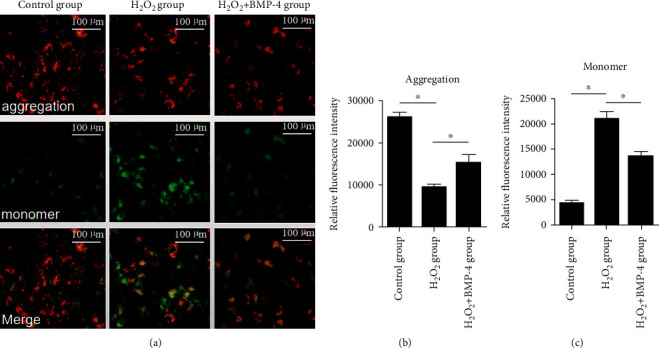
Early cell apoptosis detected by JC-1: (a) HLE-B3 cells cultured in a 96-well plate stained with JC-1; (b) fluorescence intensity quantification of aggregation (red channel); (c) fluorescence intensity quantification of monomer (green channel). Scale bars: 100 *μ*m. Data are presented as the mean ± standard deviation from three independent experiments; ^∗^*p* < 0.05.

To determine the early changes in cell apoptosis, JC-1, a fluorescent lipophilic carbocyanine dye, was used to measure mitochondrial membrane potential (*ΔΨ*m) in HLECSs. JC-1 forms complexes known as aggregates (red fluorescence) at high *ΔΨ*m. While in cells with low *ΔΨ*m, JC-1 remains in the monomeric form (green fluorescence).  Figure 5 shows the transition from red to green fluorescence. Under oxidative stress, the membrane potentials significantly decreased, and the green fluorescence intensity markedly increased. However, upon subsequent BMP-4 treatment, the red fluorescence increased significantly, indicating cells with high *ΔΨ*m.

#### 3.3.2. Effects of H_2_O_2_ Oxidative Stress on ROS Expression in HLECSs

**Figure 6:**
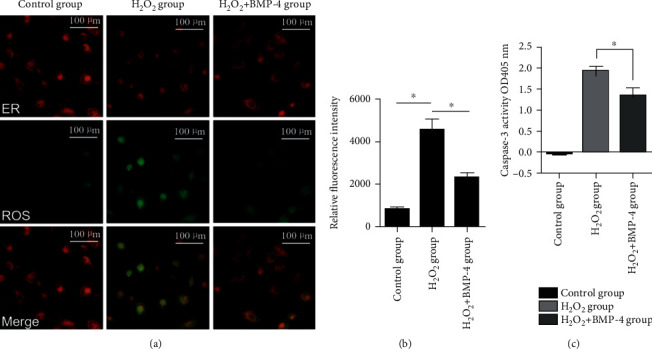
(a) The production of cellular ROS detected by DCF fluorescence. The control group showed minimal ROS production; however, in the H_2_O_2_ group, the amount of ROS, which is indicated by green fluorescence, significantly increased. The green fluorescence was attenuated in the H_2_O_2_+BMP-4 group, and the relative fluorescence intensity was significantly reduced compared with that in the H_2_O_2_ group. (b) Fluorescence intensity quantification of ROS production (green channel). (c) Caspase-3 activity in HLE-B3.

To further examine the role of H_2_O_2_ oxidative stress in HLE-B3 apoptosis, the production of ROS in cells was detected by dichlorofluorescein (DCF) fluorescence. The results demonstrated that H_2_O_2_ markedly enhanced the production of ROS; however, when BMP-4 was added, there was a significant reduction in the level of ROS (Figures 6(a) and 6(b)). Since caspases are important effector components of the cellular apoptotic pathway and activated via sequential processing of the caspase family members, we measured the expression of caspase-3 in HLECSs and observed changes in apoptosis (Figures 6(c) and 6(d)). Caspase-3 is a representative protease that plays an important role in the executive function of apoptosis. It is also the most important terminal shear enzyme in the process of cell apoptosis and one of the effectors of nuclear apoptosis. Our results demonstrated that the expression of caspase-3 in the H_2_O_2_ group was significantly higher than that in the normal control group (*p* < 0.05); BMP-4 significantly inhibited the expression of caspase-3 (*p* < 0.05).

#### 3.3.3. H_2_O_2_-Induced ER Stress Pathways Involve ATF-6 and KLF-6

**Figure 7:**
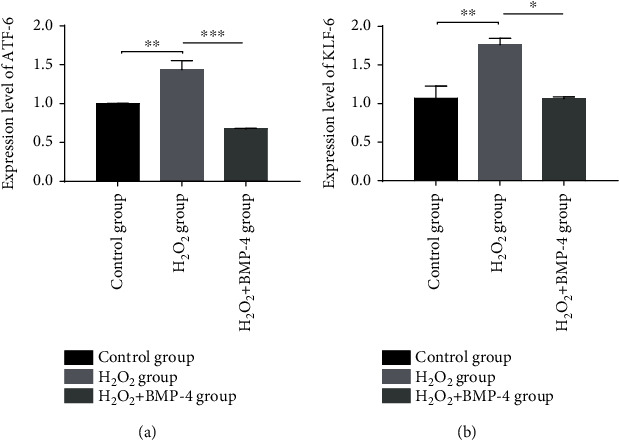
Effects of H_2_O_2_-induced oxidative stress on the expression of (a) ATF-6 and (b) KLF-6 in HLECSs. ∗ represents *p* < 0.05 vs. the H_2_O_2_ group, ∗∗ represents *p* < 0.01 vs. the H_2_O_2_ group, and ∗∗∗ represents *p* < 0.001 vs. the H_2_O_2_ group.

ATF-6 is one of the vital regulators to activate ER stress transducers and their downstream signals [10]. And KLF-6 nuclear translocations were reported to be involved in oxidative stress [11]. Therefore, the changes in ATF-6 and KLF-6 expressions in HLECSs under H_2_O_2_-induced oxidative stress were detected. The results of RT-PCR showed a significant increase in ATF-6 and KLF-6 expression under oxidative stress with respect to the control group (*p* < 0.05), and after BMP-4 treatment, the expressions of ATF-6 and KLF-6 were markedly decreased (*p* < 0.05) (Figure 7).

## 4. Discussion

Previous lens-related studies on BMPs have focused on lens development, and there is abundant evidence that BMPs, especially BMP-4, play an important role in lens induction and involvement in lens epithelial development. We investigated the effect of BMP-4 on lens epithelial cells; the results showed that BMP-4 used at different concentrations acted on HLECSs, but no significant changes were observed in cell proliferation between the BMP-4 group and the control group. Previous studies have found that the function of the BMP receptor ACVR1 is completely different in cells at different stages of lens development (i.e., promotes proliferation in the early stage and inhibits proliferation in the late stage). During the continuous development of the lens, ACVR1 plays an opposite regulatory role in cell processes, which is a novel discovery [12]. The reason for this phenomenon is not clear. It may be related to the bidirectional effect of ACVR1 on cell proliferation caused by the change in the downstream signal cascade. We speculate that BMPs play a major role in maintaining homeostasis in lens epithelial cells under normal conditions, and the effect of BMP-4 on cells may be different, depending on the cell cycle.

Apoptosis is a kind of programmed cell death, which can be observed in various types of cataracts and cultured lens epithelia during oxidative stress injury, and is the common cellular basis for the formation of noncongenital cataracts in humans and animals [13]. Many studies have confirmed that oxidative stress can lead to the cessation of cell proliferation and apoptosis [14]. In this study, H_2_O_2_ was used to establish the oxidative stress model because cataracts caused by H_2_O_2_ acting on HLECSs have been confirmed by increasing evidence [15, 16]. It has also been extensively recognized that oxidative stress is an important mediator of HLECS apoptosis, which is identified as a common molecular basis for the initiation and progression of cataracts [17, 18]. Therefore, it is important to explore protective strategies to treat or delay the development of lens opacity.

BMPs play important roles in diverse cell types, but there may be significant differences in their function depending on the organs [19]. Existing studies show that in pulmonary arteries, BMP signalling exerts important vasoprotective effects by controlling the balance between proliferation and activation of apoptosis in endothelial and smooth muscle cells [20, 21]. In contrast, BMP-4 functions as a prooxidant and prohypertensive mediator in systemic arteries [22, 23]. However, the role of BMP-4 in lens epithelial cells is unclear. In our study, Annexin V/PI double staining for detection of apoptosis revealed that apoptosis of lens epithelial cells was significantly increased when the cells were stimulated by 300 *μ*M/L H_2_O_2_, but it was significantly improved after BMP-4 treatment, indicating that treatment with BMP-4 alleviated H_2_O_2_-induced reduction of HLECS viability.

The caspase family is a group of cysteine proteases that specifically cleaves aspartic acid and plays an important role in the process of apoptosis. Studies have shown that apoptosis occurs through caspase cascade activation. In mammals, the caspase family is involved in apoptosis and contains important effector molecules of the apoptosis pathway [24]. Caspase-3 is one of the representative proteases of the caspase family, which plays an important role in the execution of apoptosis [25]. Many studies have confirmed that oxidative stress can activate caspase-3, and the antiapoptotic effect induced by the BMP signalling pathway on human pulmonary arterial endothelial cells (ECs) is in part due to the decrease in caspase-3 activity [26]. In our study, HLECSs were stimulated with BMP-4 for 2 h before and after oxidative stress, and the expression of caspase-3 decreased significantly. However, when we measured the expression of caspase-3 in cells that were only pretreated with BMP-4 under oxidative stress, there was no significant difference from that in the H_2_O_2_ group. It was speculated that the prestimulation with BMP-4 could not fully antagonize the increased reactivity of caspase-3 under oxidative stress, but continuous BMP-4 exposure could still recover some of the cell functions and reduce the expression of caspase-3. Existing research results also indicate that although the activity of caspase effectors is necessary for apoptosis, it is not enough to kill cells [27].

To examine whether the mitochondrial apoptosis pathway is involved in the inhibition of apoptosis by BMP-4, the changes in the mitochondrial membrane potential and mitochondrial apoptosis-related proteins were evaluated. The results of this study show that under oxidative stress, the changes in the mitochondrial membrane potential were markedly inhibited in HLECSs upon BMP-4 treatment. These results are consistent with those of a study reporting on antioxidant therapy against myocardial ischemia-reperfusion injury (MIRI), which also showed that antioxidants could repress the cleavage of caspase-3 [28]. Therefore, we speculate that the protective effect of BMP-4 is, to some extent, achieved by inhibiting the mitochondrial apoptosis pathway.

In addition, researchers have reported that an impaired mitochondrial electron transport chain (ETC) contributes to cardiac injury by decreasing ATP production and increasing the generation of ROS [29, 30]. In cataract-related research, ROS have long been associated with age-related nuclear cataracts and are known to adversely affect epithelial cells [31]. In this study, we found that after oxidative stress (H_2_O_2_) in human lens epithelia, intracellular ROS expression was significantly increased, indicating that under H_2_O_2_-induced oxidative stress, intracellular free radical production increased, leading to cell damage and apoptosis. Treatment with BMP-4 can significantly reduce ROS, thereby interfering with the intracellular oxidation-reduction (REDOX) balance and ER homeostasis, which leads to ER stress [32]. ER is closely connected with mitochondria through mitochondria-associated membranes (MAM) [33, 34]. ER stress can have a series of effects on cells, including damage, adaptation, and apoptosis, and can be involved in the occurrence of many diseases [35–38]. ATF-6 is an ER protein and an important transcriptional activator in ER stress. It can directly bind to the *cis*-acting original endoplasmic reticulum stress-response element (ERSE) to initiate the unfolded protein reaction (UPR) in mammals [39]. In our study, BMP-4 also significantly inhibited the H_2_O_2_-induced increase in the ATF-6 expression level. The inhibition of ER stress markers suggests that BMP-4 plays a pivotal role in ER stress and apoptosis of HLECSs. Previous studies have also shown that in myocardial ischemia-reperfusion, ATF-6 pathways play an important role in ROS-mediated ER stress. Increased ATF-6 expression in myocardial cells (*in vivo* and *in vitro)* plays a protective role against ischemia and reperfusion injuries. In transgenic mouse models, selective activation of ATF-6 can also be effective against ischemic injury in the heart [40–43].

The Krüppel-like family of zinc finger transcription factors regulates cell growth, proliferation, apoptosis, and angiogenesis [44]. KLF-6 is a ubiquitously expressed Krüppel-like transcription factor and a subfamily of DNA-binding zinc fingers involved in a diverse range of cellular processes [11]. Many studies have characterized the essential role of KLFs in maintaining homeostasis in epithelial and endothelial cells. It has been reported that KLF-6 is the first transcription factor critical to mitochondrial function under cell stress in the podocyte, and the restoration of KLF-6 attenuates mitochondrial injury and prevents cell apoptosis [45]. However, in diabetic cell damage caused by high glucose-induced oxidative stress, KLF-6 was significantly increased [46]. In our study on lens epithelial cells, incubation with prooxidants, such as H_2_O_2_, further enhanced KLF-6 expression, but it decreased upon treatment with BMP-4. Similar results have been obtained in previous studies on primary hepatocytes. KLF-6 expression increases with high levels of cytochrome P450 2E1 (CYP2E1); however, antioxidants and CYP2E1 inhibitors prevent this increased expression of KLF-6 [39]. Thus, KLF-6 has many diverse functions; the regulation of KLF-6 under cell stress and how KLF-6 senses ROS need to be further explored.

## 5. Conclusions

The functions of BMP-4 in lens epithelial cells under oxidative stress have not been previously elucidated. Herein, we provide the first evidence that BMP-4 alleviated H_2_O_2_-induced oxidative stress and apoptosis in HLECSs, which is associated with the ER stress/mitochondria-mediated caspase-dependent apoptosis pathway. The causes of cataracts are multifactorial, and the pathogenesis is complex. Many studies have shown that the abnormal metabolism and injury of lens epithelial cells caused by thermal radiation, ultraviolet radiation, H_2_O_2,_ and other stress conditions are closely related to the occurrence of cataracts. BMP-4 can effectively inhibit the apoptosis of lens epithelial cells under oxidative stress. These findings might be important for understanding the role of BMPs in cataracts and may provide novel insight into the early prevention and control of cataracts.

## Figures and Tables

**Table 1:** The sequences of the primers.

No.	Primer	Sequences (5′ to 3′)
1	ho ATF-6 U	TCAGCCCAAGCCTTTATTGC
2	ho ATF-6 D	TGATGGTTTTTGCTGGAACACT
3	ho KLF-6 U	GGTCAGCTCGGGAAAATTGA
4	ho KLF-6 D	CCTGCTCAGTTCCGGAGAAG

## Data Availability

The data used to support the findings of this study are available from the corresponding author upon request.
